# Desmoplastic Fibroma of the Mandible: A Case Without Recurrence After Enucleation

**DOI:** 10.7759/cureus.42213

**Published:** 2023-07-20

**Authors:** Nurul Izyan Zainuddin, Lim Chin Kai, Daniel Lim, Tilakaratne WM

**Affiliations:** 1 Department of Oral and Maxillofacial Clinical Sciences, Faculty of Dentistry, Universiti Malaya, Kuala Lumpur, MYS

**Keywords:** bone benign tumor, bone tumors, desmoplastic fibroma, oral surgery, oral and maxillofacial pathology

## Abstract

Desmoplastic fibroma (DF) is a rare, benign, yet locally aggressive bone tumor. It frequently affects the facial bones, and the mandible is the most commonly affected site. Treatment of choice is the removal of the tumor with resection of surrounding bone due to its aggressive behavior. We report a case of DF where the tumor showed resolution and almost complete bone deposition following enucleation. Although DF has a high recurrence rate, the patient remains disease-free 31 months post-surgery.

## Introduction

Desmoplastic fibroma (DF) is an uncommon bone tumor, accounting for only 0.1% of all bone tumors. According to the WHO classification, although classified as benign, it usually demonstrates aggressive behavior with a moderate to high recurrence rate. Facial swelling, tooth mobility, and extensive bone destruction are the findings commonly observed in patients with DF [1]. DF primarily affects the pelvis, femur, radius, and tibia; in the facial region, it is most commonly found in the mandible [2,3,4]. DF is also known as the intraosseous counterpart of desmoid-type soft tissue fibromatosis. Diagnosing DF can be challenging as the tumor can resemble a variety of benign and malignant tumors from their radiological and histopathological presentations, sharing almost identical characteristics of soft tissue fibromatosis at a microscopic level. This article presents a case of DF of the mandible, discussing its management, histopathological features, immunohistochemical findings, and treatment outcome. Although resection is the preferred treatment option due to the possibility of recurrence, the patient consented to enucleation of the tumor only.

## Case presentation

**Figure 1:**
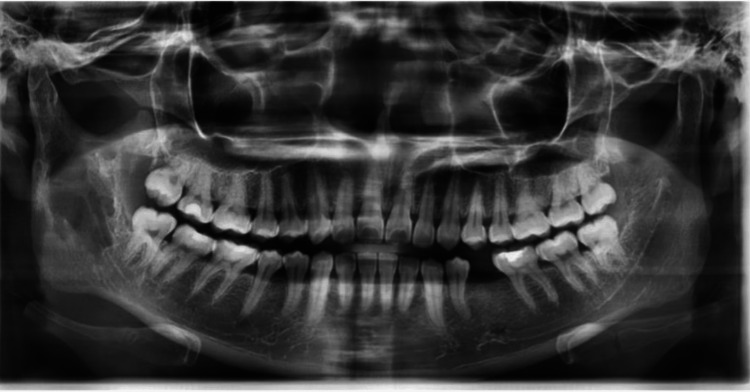
Dental panoramic tomograph (DPT) reveals multilocular radiolucency with ill-defined margins at the right ramus of the mandible.

A 28-year-old female patient was referred to the oral and maxillofacial surgery clinic by a private general dentist. The patient had been experiencing recurrent pain and swelling in the right mandible for the past month and numbness in the area for the past four years. Upon initial presentation at the clinic, there was no visible extraoral swelling. Intraorally, a slight soft tissue swelling was noted on the medial aspect of the right ramus. It was soft in consistency. An initial cone-beam computed tomography (CBCT) scan taken by the referring clinic revealed the presence of a 1.8 cm x 3 cm x 2 cm mass with an irregular margin in the right mandibular ramus. The overlying mucosa was normal. The dental panoramic tomograph (DPT) revealed a multiloculated, irregular radiolucency on the right side of the mandible, extending from the ramus to the coronoid notch region. No tooth was detected within the cavity (Figure 1). An exploratory excisional biopsy under general anaesthesia was planned.

**Figure 2:**
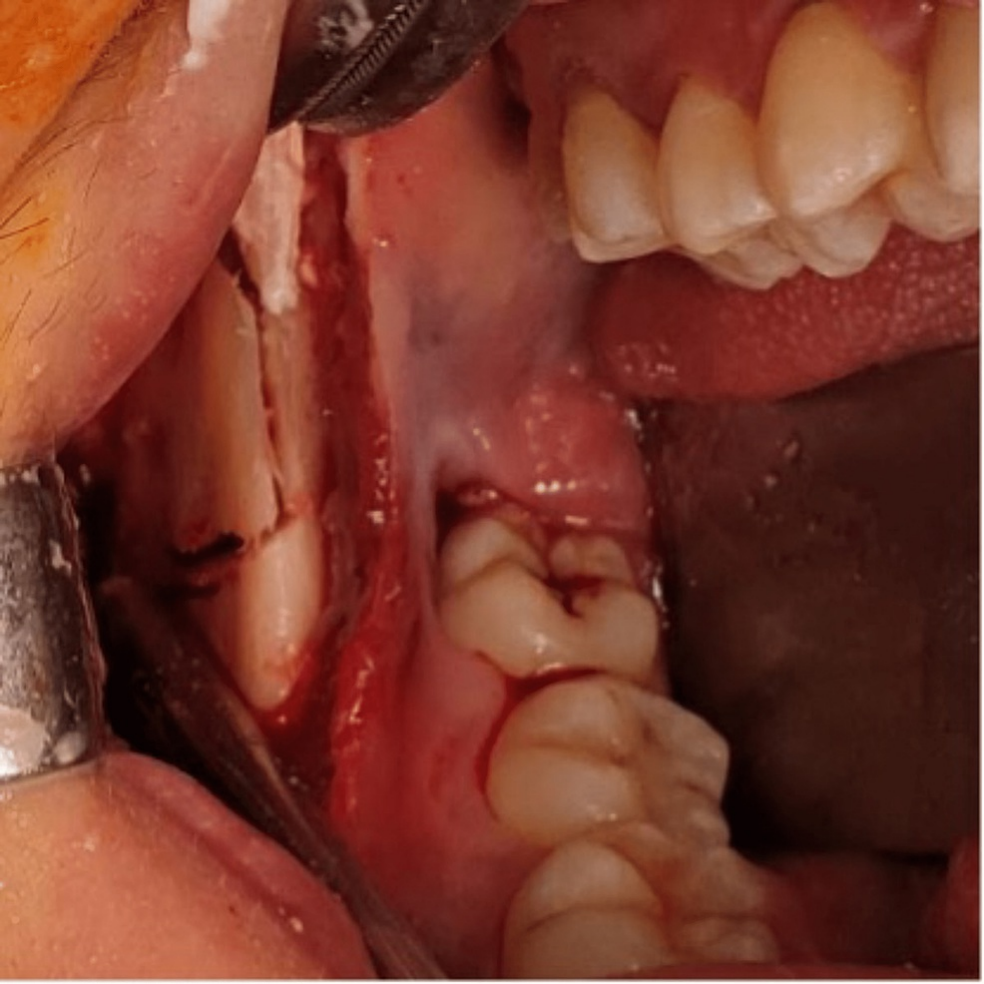
Intraoperative photograph showing the elevation of the buccal bone lid to expose the tumor.

During the surgery, a vestibular incision was made along the external oblique extending to the anterior aspect of the ramus. A buccal bone lid window was created, exposing the tumor cavity (Figure 2). The bone lid cut surface was beveled to facilitate its placement back after tumor removal. The entire tumor was successfully excised, and the underlying bone was carefully curettaged, followed by thorough irrigation with normal saline. Varnish ribbon gauze soaked in the white head was placed in the cavity for three minutes. The cavity was then flushed with normal saline, and the inner surface of the bone lid was also curettaged and cleaned before being placed back to cover the cavity. The flap was approximated, and the surgical site was closed primarily. Surgical resection was not performed as per the patient's request. The excised specimen was sent to the oral pathology laboratory for histopathological examination. Ameloblastoma, aneurysmal bone cyst, and intraosseous squamous cell carcinoma were among the differential diagnoses.

**Figure 3:**
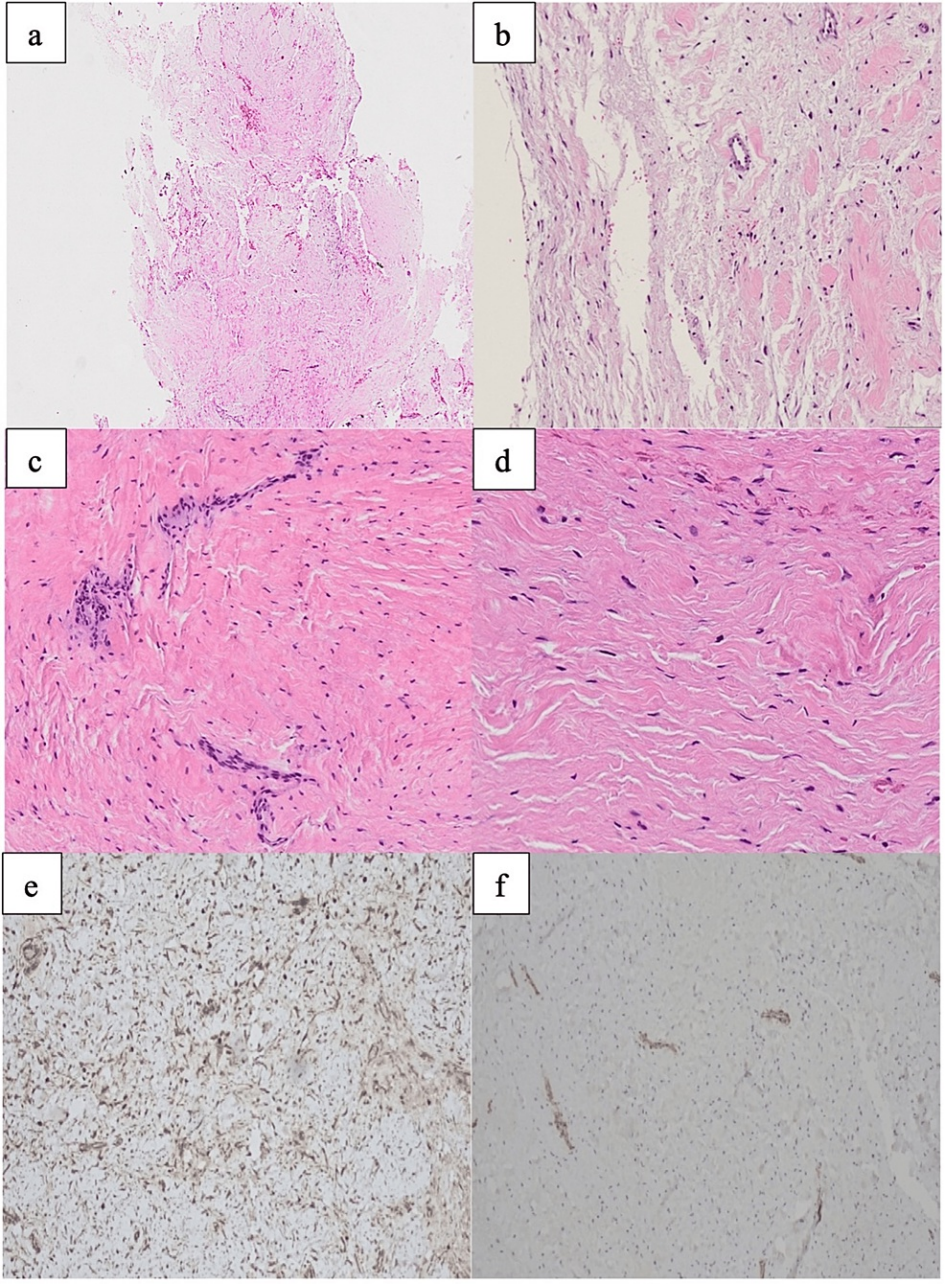
Photomicrograph of low magnification from the incisional biopsy sample of the tumor. (a and b) The periphery of the lesion reveals no encapsulation but shows myxoid change as it extends to the periphery. (c and d) The tumor showing coarse, densely packed collagen fibers with uniform spindle cells with tapering nuclei. Cellular atypia was not evident. Mitosis is not observed. (e) Diffuse positivity is observed with vimentin. (f) Equivocal positivity is observed with SMA. SMA: Smooth muscle actin.

The histopathological findings revealed multiple fragments of dense fibrous connective tissue containing spindle and stellate-shaped fibroblasts of low to moderate cellularity without significant cytological atypia. The stroma was predominantly collagenous, with a few myxoid areas observed. No mitosis was observed. Fragments of vital lamellar bone, vascular channels, and regions of hemorrhage were also evident. The specimen did not exhibit any evidence of odontogenic epithelium. Immunohistochemical studies were conducted using vimentin, smooth muscle actin (SMA), and desmin. Immunopositivity was only observed with vimentin, SMA results were equivocal, and desmin showed negative results (Figure 3). The case was diagnosed as "intraosseous desmoplastic fibroma."

Follow-up

**Figure 4:**
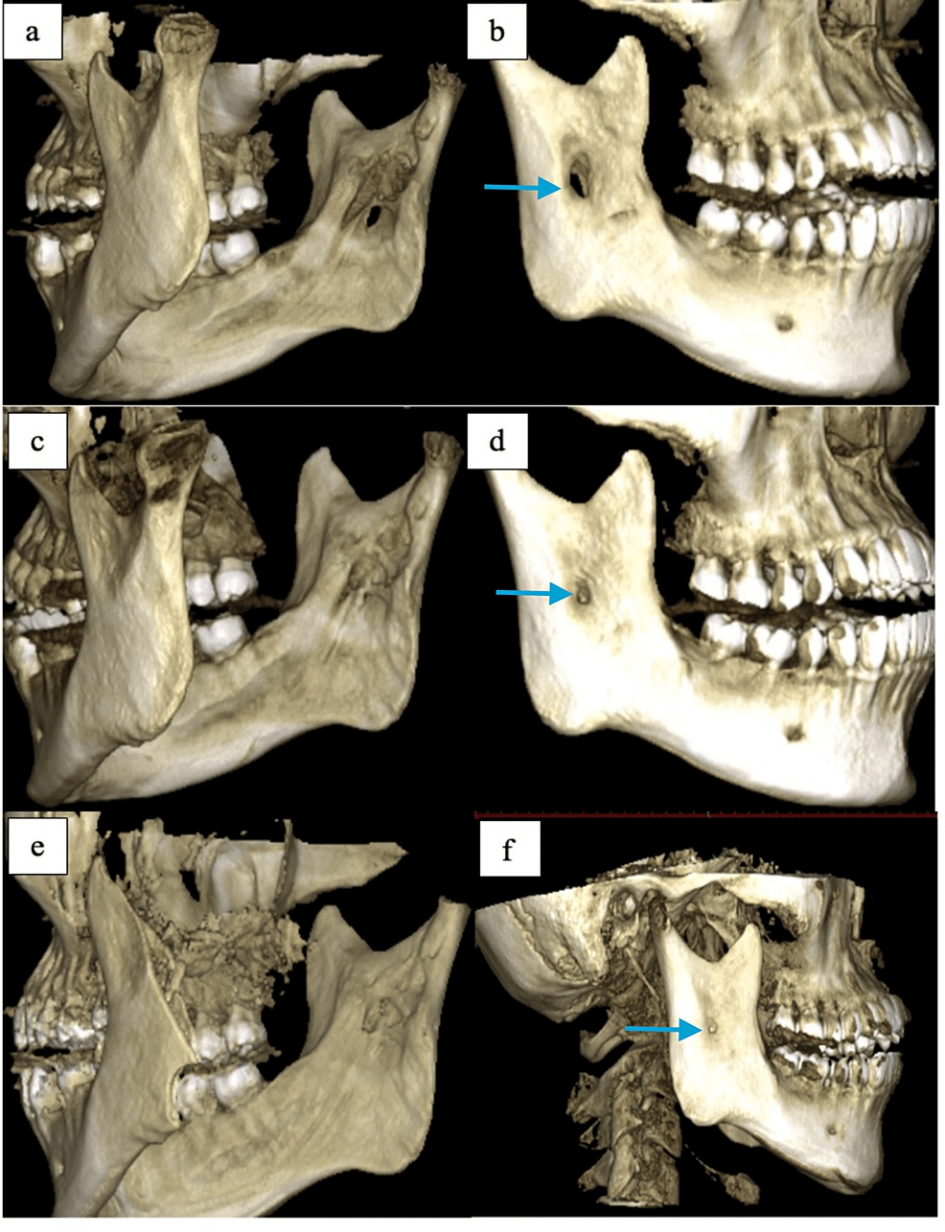
Arrow depicts the evidence of gradual bone deposition during the patient's review appointments. (a and b) CBCT scan of the mandible taken during the six-month post-operative review showing gradual deposition of bone in the cavity. (c and d) Marked gradual bone deposition filling the cavity after 19 months. (e and f) Almost complete bone deposition 31 months after the surgery.

During the six-month post-operative review, the DPT and CBCT images showed a reduction in the cavity size, with a remaining buccal perforation at the right ramus. Another exploratory biopsy performed in the region during this review revealed granulation tissue with no evidence of tumor cells. The patient's recovery was uneventful, and the surgical site healed. The patient reported mild hypoesthesia post-surgery, which subsequently resolved at the six-month post-operative review. During the 19-month post-operative review, the CBCT scan showed no evidence of recurrence. The buccal cavity exhibited a marked reduction in size, and the perforation from the lingual to the buccal region was no longer observed. No swelling was noted intra-orally upon palpation. During her recent review 31 months after the surgery, almost complete bone deposition was noted (Figure 4). A periodic six-month follow-up appointment was planned for the next three years.

## Discussion

DF, originally identified by Jaffe in 1958, is characterized as a fibrous tumor consisting of fibroblasts and collagen fibers resembling an abdominal desmoid tumor. In 1965, Griffith and Irby documented the initial instance of DF affecting the facial bone [5]. According to the most recent classification of head and neck tumors by the WHO, DF is defined as a locally aggressive fibroblastic/myofibroblastic tumor comprising benign spindle cells surrounded by a collagenous background that resembles desmoid-type fibromatosis. [1] Approximately 75% of patients with DF are under 30 years old, with a mean age of 16 years. Although any bone can be affected, the mandible (22%) is the most commonly affected, followed by the femur (15%), pelvic bones (13%), radius (12%), and tibia (9%). The mandible accounts for 82% of all cases occurring in the facial region and tends to involve a more posterior location, as seen in our case. In contrast, the remainder involves the maxilla, usually in the anterior region. There is a slight female predilection [1,4]. Most patients with DF present with asymptomatic swelling or facial asymmetry. Patients may occasionally complain of restricted mouth opening, teeth mobility, infection, and bleeding [1].

Radiographic features

Radiographic features of DF are non-specific. A unilocular or multilocular, well-defined or irregular radiolucency with variable expression of marginal sclerosis are among the presented radiographic features. Therefore, DF often mimics other common as well as unusual pathologies of the jaws, including ameloblastoma, odontogenic myxoma, aneurysmal bone cyst, chondromyxoid fibroma, central hemangioma, and dentigerous cyst [6,7]. Two more unusual patterns that have been described are periapical pathology in relation to an endodontically treated tooth and an interradicular position at presentation. The sunray appearance has also been reported in some cases. This feature, mimicking osteogenic sarcoma, may be particularly noteworthy because it may incorrectly result in a diagnosis of malignancy [8]. Furthermore, spindle cell histology combined with rapid growth and bone destruction frequently seen with DF may result in an incorrect diagnosis. CT and MRI are important for accurate surgical care and planning. CT allows the demonstration of cortical perforation and margins, while MRI helps to show the separation of the intraosseous tumor from the bone by displaying the margins clearly [9].

Histopathological features

The histopathological features of DF show mature, dense, and collagenous fibrous connective tissue with bland spindle and stellate fibroblasts. Cellular atypia, mitosis, and necrosis have not been reported. Several entities show histopathological resemblance to DF. Amongst those are fibrous dysplasia, central odontogenic fibroma, and low-grade fibrosarcoma. The histopathological features of fibrous dysplasia show trabeculae of viable bone surrounded by low to moderate cellular fibrous connective tissue. The diagnosis becomes more complicated when only small samples with a few bone fragments are submitted. Due to the infiltrative nature of DF, it is common to visualize entrapped bone fragments within the tissue sections. In our case, the submitted sample was sufficient to exclude fibrous dysplasia as the bony trabeculae evident in the sections did not resemble those of fibrous dysplasia. Central odontogenic fibroma shows mature, variably cellular fibrous connective tissue with scattered bland, odontogenic islands within the tissue. In our case, few areas containing clusters of cells resembling odontogenic islands were present. However, these areas showed positivity with the SMA marker, making this diagnosis unlikely. It is essential, albeit difficult, not to confuse DF with low-grade fibrosarcoma, as the latter is a malignant neoplasm. In comparison to DF, the histopathological features of fibrosarcoma are more cellular, with plumper cells and a distinct herringbone growth pattern [10]. DF, on the other hand, has a more unidirectional pattern of spindle cells and nuclei without atypia. The distinction between these two entities can only be detected when metastasis or recurrence occurs. The presence of myxoid areas in this case also raises the possibility of low-grade fibromyxoid sarcoma (LGFS). This tumor shows alternating fibrous and myxoid areas containing bland spindle or stellate cells in a whorled growth pattern. Due to its bland morphology, LGFS can be difficult to distinguish from other benign low-grade sarcomas. However, MUC4 has been proven to be highly sensitive and specific in diagnosing LGFS [11]. Currently, there is no immunohistochemistry (IHC) marker considered pathognomonic in diagnosing DF; hence the diagnosis of DF is often made by exclusion. However, several IHC markers such as vimentin (92%), SMA (80%), muscle-specific actin (63%), S100 (7%), low Ki-67 expression, and lack of nuclear ß-catenin expression have been routinely used to confirm the origin of this tumor and to exclude other lesions mimicking DF [12]. Vimentin, SMA, and desmin were used for the present case, and only vimentin showed strong positivity with equivocal positivity for SMA. Other differential diagnoses, such as a desmoid tumor, show nuclear ß-catenin positivity and CTNNB1 or APC mutation, while fibrous dysplasia reveals GNAS gene mutation. Low-grade fibrosarcoma reveals MDM2, CDK4, and SATB2 positivity. In some cases, low-grade myofibroblastic sarcoma can also have a histopathological resemblance to DF, and this can be confirmed with SMA, desmin, and nuclear ß-catenin positivity in this tumor, but not in DF. Other tumors, such as synovial sarcoma, can be distinguished from DF as it shows TLE1 positivity and SS18 rearrangement, while spindle cell rhabdomyosarcoma of the jaw shows TFCP2 rearrangement. Myoepithelial tumors reveal S100 and CAM 5.2 positivity, INI1 loss, and EWSR1 rearrangement [1].

Treatment modalities and prognosis

A review by Woods TR et al. in 2015 reported that 109 cases with recorded treatment modalities were available for review. Among the data, the most common treatment was en bloc resection (58%, 63/109), followed by excision/enucleation (40%, 44/109), and curettage alone (12%, 13/109). Chemotherapeutic agents, such as vincristine, doxorubicin (Adriamycin), and dacarbazine (DTIC), were also used in 7% of cases (8/109). Follow-up information in their study was available for 120 cases. Recurrence was reported in 58% of the 120 cases. The follow-up time periods ranged from 3 months to 17 years, with an average follow-up time of 3.3 years [12]. Said-Al-Naief N et al. (2006) reported recurrence rates following excision and enucleation ranging from 20% to 40% and curettage as high as 70% [4]. In a separate report by Karimi A et al. in 2020, the recurrence rate was about 40% to 47% for lesions treated by curettage or intralesional resection [13]. However, due to its aggressive behavior and high recurrence rate, many authors recommend complete surgical resection of the lesion with safety margins. It has been noted that tumors with high cellularity tend to recur more often than those with lower cellularity [12]. Some authors reported that tumors exhibiting cortical perforation, soft tissue involvement, and moderate to high cellularity should be treated by wide resection with a safe margin. Although wide resection is highly recommended, Nishida J et al. reported five patients treated with aggressive curettage alone without recurrence during a follow-up of 5-9 years [14]. Another treatment modality, such as radiotherapy, has been utilized in treating DF and has shown tumor resolution [15]. However, as DF is a benign tumor, such aggressive treatment is more opted for managing malignancy cases. It has also been mentioned that malignant transformation is rare in the case of DF [16]. Radiotherapy carries the risk of various complications and side effects, and since DF typically affects the mandible, which is a vital structure for chewing, speaking, and facial aesthetics, the potential harm caused by radiation may outweigh the benefits. DF is also characterized by its tendency to aggressively invade and damage the adjacent bone tissue. Thus, it may not effectively address the infiltrative nature of DF or prevent its recurrence, although it can effectively target cancer cells [17]. Radiotherapy, as well as chemotherapy, thus have been suggested only in cases where the behavior of the tumor and especially when complete tumor resection is impossible [18]. Surgery is considered the primary treatment approach, aiming to remove the tumor and the affected bone. In our case, the patient had consented to enucleation only, and no recurrence has been demonstrated following treatment with enucleation up to 31 months post-surgery. 

## Conclusions

DF is an uncommon, locally aggressive benign bone tumor that typically resembles numerous facial bone tumors due to its radiological presentation. Clinicopathological and radiographic correlations are essential to achieve a definitive diagnosis. The treatment of choice is surgical excision with a wide margin due to its high recurrence rate. Recurrence after resection is approximately 10%, and chemotherapy may be considered if excision is not feasible. However, radiotherapy is not recommended. Enucleation and curettage may have a therapeutic role in carefully selected cases.
